# Deformation Behavior and Processing Maps of 7075 Aluminum Alloy under Large-Strain Thermal Compression

**DOI:** 10.3390/ma16237432

**Published:** 2023-11-29

**Authors:** Erli Xia, Tuo Ye, Sawei Qiu, Limei Liu, Fang Luo, Huanyu Yue, Yuanzhi Wu

**Affiliations:** 1 Research Institute of Automobile Parts Technology, Hunan Institute of Technology, Hengyang 421002, China; 2021001046@hnit.edu.cn (E.X.);; 2 School of Intelligent Manufacturing and Mechanical Engineering, Hunan Institute of Technology, Hengyang 421002, China; 21010240108@xs.hnit.edu.cn; 3 School of Material Science and Engineering, Hunan Institute of Technology, Hengyang 421002, China

**Keywords:** 7075 aluminum alloy, large-strain thermal deformation, processing map, microstructure observation

## Abstract

The investigation of thermal deformation behavior plays a significant role in guaranteeing the overall performance of alloy materials. In this manuscript, a series of isothermal compression tests at different temperatures (300, 350, 400, and 450 °C) and strain rates (0.001, 0.01, 0.1, and 1 s^−1^) were conducted to study the thermal deformation behavior of 7075 aluminum alloy. Subsequently, processing maps at a strain from 0.4 to 1.39 were established according to the stress–strain data obtained from various deformation parameters. The microstructural evolution of the target alloy was observed with an optical microscope and transmission electron microscope. The results reveal the unstable regions are located at (360–450 °C, 0.04–1 s^−1^) and (300–315 °C, 0.01–0.22 s^−1^). Precipitation particles, pinned dislocations, and highly dislocated areas can be observed in the microstructure of the alloy in the unstable regions. This is a potential crack and defect formation point. The identified optimum processing parameters are located at (375–450 °C, 0.001–0.03 s^−1^), with a maximum dissipation efficiency of 0.6.

## 1. Introduction

Because of their excellent mechanical properties [1,2], Al-Zn-Mg-Cu alloys are manufactured as structural parts, which are extensively used in the aerospace, petroleum, automobile, and railway transportation industries [3,4]. The thermal deformation process has a decisive effect on the mechanical properties of an alloy. Plenty of studied findings [5,6,7] have proven that thermal deformation parameters influence the microstructure, and the microstructure of alloys determines the ultimate performance of alloys. Thus, in order to achieve the desired properties of Al-Zn-Mg-Cu alloys, the study of thermal deformation characteristics and the identification of the optimum processing parameters are of great importance.

Founded on the dynamic material model (also abbreviated as DMM), Prasad and his colleagues proposed the processing map theory [8,9]. This can be constructed by superimposing a power dissipation map upon an instability map. As a reliable industrial application tool for avoiding material defects and ensuring excellent material properties, the processing map was extensively adopted to investigate thermal workability, obtain the optimum processing parameters, and control the microstructure of metals and alloys [10,11,12]. In order to acquire the unstable region, Wu et al. [13] considered the negative dissipation efficiency. The processing map of Al-Zn-Mg-Er-Zr alloy under thermal compression was constructed, and the thermal deformation regions owning a high power dissipation efficiency were identified. Long et al. [14] took Ti-6Cr-5Mo-5V-4Al alloy as the research object. The thermal deformation characteristics and processing map were investigated. The results showed that the optimum processing domain was located at (757–840 °C, 0.001–0.032 s^−1^). To improve the thermal workability of the 2014 forging aluminum alloy, Li et al. [15] conducted compression tests and established the processing map. Finally, the optimum processing parameters were obtained through the analysis of the processing maps. The thermal deformation characteristics of 2219 aluminum alloy were investigated by He et al. [16]. Via processing maps, the optimum processing parameters were determined, which were located at 380–510 °C and 0.008–0.78 s^−1^. The thermal deformation behavior of 6000 series aluminum alloy was reported in references [17,18], and the three-dimensional processing map was employed to explore the thermal workability of aluminum alloy. In addition, the literature findings reveal that certain relations exist between the dissipation efficiency and dynamic softening mechanisms. The thermal deformation characteristics of Al–Zn–Mg–Cu alloy were investigated by Shi et al. [19]. By taking advantage of the processing map and the microstructure observation of dissipation efficiency, they found that at a lower dissipation efficiency, the dominant softening mechanism is discontinuous dynamic recrystallization; at a higher dissipation efficiency, continuous dynamic recrystallization predominates.

The processing map, as an effective tool, has been widely adopted in 7000 series aluminum alloy for investigating its hot workability and obtaining the optimum processing parameters. Jin et al. [20] conducted a series of tensile tests to study the deformation characteristics of 7050 aluminum alloy. The processing map and the microstructural observation method were utilized to explore the hot workability of 7050 aluminum alloy. The results display that the stable domain is situated at (410–460 °C, 0.0001–0.001 s^−1^). The deformation behavior and hot workability of 7075 aluminum alloy (abbreviated as AA7075 thereinafter) were investigated via isothermal compression tests under different deformation parameters [21]. The results show that a relatively homogeneous microstructure can be obtained when manufacturing in the parameter region of (350–450 °C/0.001–0.05 s^−1^). After the isothermal compression test, the authors of reference [22] established the processing map for improving the workability of as-cast AA7075. The processing map and microstructure analysis results show that the most appropriate processing window is situated at (425–465 °C, 0.01–1 s^−1^). By considering the strain effect, Luo et al. [23] constructed a constitutive equation for Al-Zn-Mg-Cu alloy under different deformation parameters. The processing maps at two strains (0.3 and 0.6) were analyzed. With the assistance of microstructural observation, the optimum hot processing parameter was determined at (390–440 °C, 0.010–0.316 s^−1^).

During the hot working process, the formation of a microstructure is sensitive to the deformation temperature, strain rate, and strain level. Additionally, the macroscopic properties of the materials are decided according to the microstructure of the materials. Thus, the selection deformation parameter is a key procedure in the material working process [24,25]. According to the above thermal deformation research on aluminum alloys, the true strain adopted in the compression tests is usually less than one. However, under large-strain compression conditions, the microstructural evolution and flow behavior of the alloys may exhibit different trends. Correspondingly, the processing map and the optimum working parameters will be different from those in small-strain thermal compression conditions. And the working parameters greatly affect the properties of the materials during the working process. Thus, the investigation of thermal compression behavior and the processing map under a large strain is of great importance for gaining the final properties of the component.

In the present manuscript, the stress and strain characteristics of AA7075 under isothermal compression tests with different parameters are studied. In order to construct the processing map, the strain rate sensitivity, power dissipation efficiency, and instability parameters are evaluated. Subsequently, using the DMM, processing maps are established. Optical microscope (OM) and transmission electron microscope (TEM) observations are employed to analyze the microstructure evolution and determine the hot working parameters of the target alloy.

## 2. Materials and Experimental Methods

In this manuscript, a typical Al–Zn–Mg–Cu alloy, namely AA7075 (Metal Materials Co. LTD, Shenzhen, China), was selected as the research object. The chemical composition of the aluminum alloy is given in Table 1. The studied AA7075 is an extrusion rod, which has a radius of 17.53 mm.

**Figure 1:**
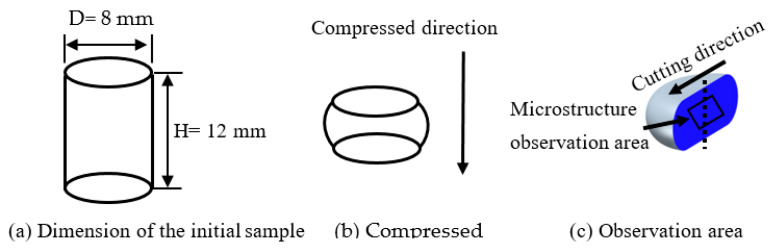
Sketch map of the sample and observation area.

The cylindrical sample was obtained for isothermal compression tests. Its dimensions are displayed in Figure 1a. The extrusion rod was machined into cylindrical samples of 8 mm in diameter and 12 mm in height for the isothermal compression tests, as illustrated in Figure 1a. The extrusion direction and compression direction of the sample were parallel. Using a Gleeble-3500 thermomechanical simulator (which was made by DSI Corporation, in St. Paul, MN, USA), isothermal compression tests were performed at four distinct strain rates (0.001, 0.01, 0.1, and 1 s^−1^) and temperatures (300, 350, 400, and 450 °C). The specimens were heated at a 10 °C/s rate to the predetermined deformation temperature before the test started. They were then held at the tested temperature for 180 s to remove the thermal gradient. The sample was compressed to a 75% reduction for all the deformation conditions (the corresponding true strain was approximately 1.39), as shown in Figure 1b. In order to reduce the friction effect during the compression test, graphite was used as the lubricant between the contact surfaces of the press head and the samples. A computer was employed to record the experimental data. In order to retain the microstructure of the sample after compression, the samples were quenched in water as soon as the compression tests had been finished.

In order to explore the effect of the deformation parameters on microstructure evolution, OM and TEM observation methods were used. After the thermal compression test, all the samples were cut along the compressed direction containing the axis surface, and the center area of the cutting surface was selected as the microstructure observation region, as depicted in Figure 1c. For OM observation, the samples were polished with a polishing machine (Ortlay, Tianjin, China), and then corroded in 4% HBF_4_ + 96% H_2_O solution (Runfeng Petrochemical Co., LTD, Nantong, China). The observation was conducted using an Axio Vert A1 CarlZeiss optical microscope, which was produced by GmbH in Oberkochen, Germany. The samples intended for TEM observation were mechanically reduced to a thickness of 80 μm, and subsequently, using Tenupol-5 electro-polishing apparatus (Struers, Shanghai, China), they were thinned with a twin jet in 30% HNO_3_ and 70% CH_3_OH solutions (Runfeng Petrochemical Co., LTD, Nantong, China) at a temperature of −25 °C. The microstructures were observed under a Tecnai G2 20 TEM. This was provided by FEI Company from the Hillsboro, OR, USA.

## 3. The Theory for Construction of Processing Map

In order to improve the hot workability of alloy materials, a processing map was developed, which was founded on the DMM theory [26,27]. The DMM theory assumes that, during the hot working process, the total consumed energy *P* is composed of two parts, which can be expressed as:

(1) $$P=\sigma \dot{\varepsilon }=\int_{0}^{\dot{\varepsilon }}\sigma d\dot{\varepsilon }+\int_{0}^{\sigma }\dot{\varepsilon }d\sigma$$

here, *σ* represents the flow stress, and $\dot{\varepsilon }$ signifies the strain rate; *G* denotes the power dissipated by deformation heating and plastic deformation; *J* signifies the power consumed by microstructural evolution, such as dynamic recovery, super-plastic flow, dynamic recrystallization, and so on.

It is commonly accepted that, during the hot working process, the strain rate has a great effect on the flow stress level. In order to explore the energy dissipated status, the strain rate sensitivity coefficient (*m*) is first introduced to characterize the hot working process. *m* can be obtained by differentiating the power between the deformation heat and microstructural evolution:

(2) $$m=\frac{dJ}{dG}=\frac{\partial P}{\partial G}\frac{\partial J}{\partial P}=\frac{\dot{\varepsilon }d\sigma }{\sigma d\dot{\varepsilon }}=\frac{\partial (\ln\sigma )}{\partial (\ln\dot{\varepsilon })}$$

Utilizing the strain rate sensitivity coefficient, the flow stress during the hot working process can be expressed as:

(3) $$\sigma =K^{\dot{\varepsilon }m}$$

where *K* is a constant. Substituting Equation (3) into Equation (1), the value of *J* can be obtained as:

(4) $$J=\frac{m}{m+1}\sigma \dot{\varepsilon }$$

At the condition of linear dissipation, *m* is equal to 1. In this situation, the value of *J* is the max.

(5) $$J_{\max}=\frac{1}{2}\sigma \dot{\varepsilon }$$

However, during the hot working process, it cannot be regarded as a linear dissipater. The power dissipation efficiency *η* is defined as the ratio of *J* to *J*_max_:

(6) $$\eta =\frac{J}{J_{\max}}=\frac{2m}{1+m}$$

*η* signifies the power dissipation efficiency of the alloy materials during the thermal deformation process. The dissipation efficiency at various deformation conditions can be calculated by utilizing Equations (2) and (6), so as to construct a visualized power dissipation map. There exists a certain relationship between *η* and deformation mechanisms that can reflect the workability of materials. Thus, during the construction of the processing map, *η* occupies an important position.

Moreover, to avoid the occurrence of cracks and flow localization, the calculation method of the instability parameter was also presented by Prasad and Seshacharyulu [9], which can be used to identify the instability area. It can be expressed as:

(7) $$\xi (\dot{\varepsilon })=\frac{\partial \ln[m/(m+1)]}{\partial \ln\dot{\varepsilon }}+m<0$$

From Equation (7), it can be seen that the value of *ξ* alters with the deformation parameters. By calculating *ξ* under different temperatures and strain rates, a visualized instability map can be established. On the instability map, the domain with negative *ξ* values signifies flow instability. The thermal workability of a material will be significantly deteriorated when processing in the flow instability region. Working in the instability regions, the microstructure of alloys tends to show localized flow, cracks, and other deformed defects. Therefore, during the structural component manufacturing process, the hot working parameter should not be located in those zones.

For better guidance in the selection of hot processing parameters, the power dissipation map is combined with the instability map to obtain the processing map. Abundant documents have proved that the processing map is capable of finding the optimum processing parameters. Additionally, it can establish a certain relationship between the power dissipation efficiency and the deformation mechanisms. Therefore, the processing map can be a useful tool for guiding the metalworking and controlling of alloy microstructures.

## 4. Results and Discussion

### 4.1. Initial Microstructure

**Figure 2:**
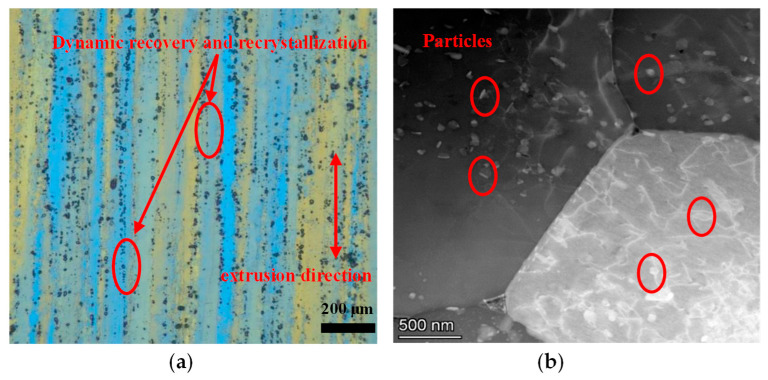
The initial microstructure of 7075 aluminum alloy: (**a**) Optical microscope (OM) observation; (**b**) transmission electron microscope (TEM) observation.

The initial microstructure of AA7075 before the isothermal compression test is displayed in Figure 2. It exhibits highly elongated, fibrous grains, with the direction of the grain parallel to the extrusion direction. The extruded bar was air-cooled during production. Hence, some recovery and recrystallization structures can be observed. Furthermore, there are evenly distributed particles in the grain structure.

### 4.2. The True Stress–strain Curve

**Figure 3:**
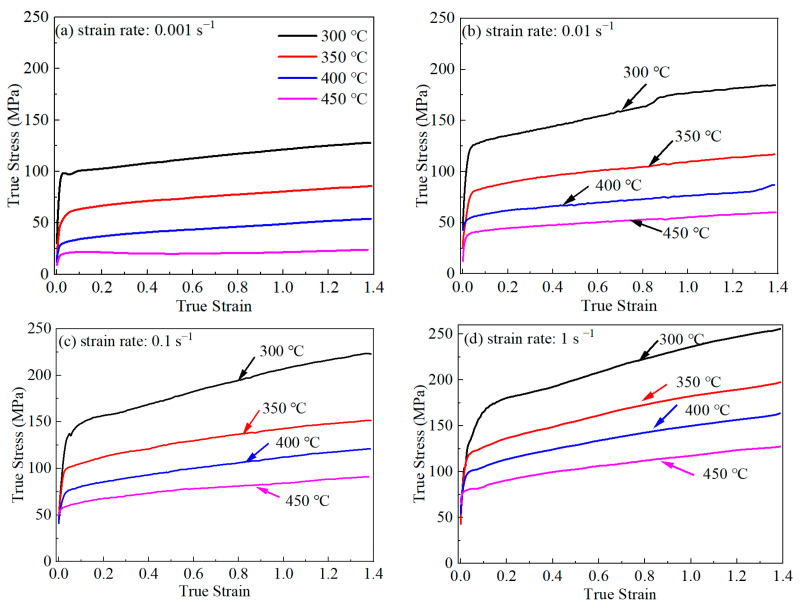
The true stress–strain curves of AA7075 at different compressed conditions.

After the processing of the engineering stress–strain data, the true stress–strain curves of AA7075 from the compression tests can be obtained. The stress–strain curves under different parameters are presented in Figure 3. At the initial deformation stage, the flow stress increases rapidly with the strain growth. During the hot working process, work hardening and dynamic softening effects co-exist. Deformation induces the propagation of dislocation, and it shows a work-hardening phenomenon. On the other hand, internal energy growth will trigger dynamic recovery and recrystallization, which shows a dynamic softening effect on the macroscopic scale. At this stage, the rapid growth of the dislocation density is responsible for the increase in true stress. In other words, the work-hardening effect far surpasses the effect of softening at this deformation stage. As deformation progresses, when the instantaneous flow stress exceeds the yield strength, a typical plastic deformation process is observed. At this deformation stage, the dynamic softening effect is gradually enhanced. It partially counteracts the effect of work hardening, and as a consequence, the growth of the stress becomes slower in the subsequent deformation process. The relationship between softening and work hardening decides the characteristics of the flow stress–strain curves [28]. According to the above analysis, this is manifested in the studied alloy, where the softening effect is smaller than the hardening effect [29]. Thus, the flow stress exhibits a slightly upward trend in the following deformation process.

In addition, it can be discovered from Figure 3 that the stress level of AA7075 is highly dependent on the compressed temperature and strain rate. The compressed temperature has a negative effect on the flow stress. This is mainly due to the fact that, at higher temperatures, the dislocations in the crystals own more energy and become more active. They can overcome the energy barrier, and the rearrangement of dislocations will happen. As a result, the stress is decreased. On the other hand, the strain rate has a positive effect on the true stress. At higher strain rates, dislocation movement and annihilation are not sufficient because of the fast deformation rate. Thus, the stress increases with the strain rate. The stress–strain characteristics of AA7075 under thermal compression conditions are similar to others’ findings [30]. Generally, a low flow stress level signifies that the alloys tend to deform. In this situation, the pinning effect and stress concentration are relatively minor. The processed component has an even, free-of-defects microstructure and has superior mechanical properties. Therefore, the optimum processing domain is usually situated in the low-level flow stress area. Considering the effect of temperature and strain rate on the flow stress, it can be speculated that the high temperature and low strain rate areas are conducive to obtaining the optimum processing parameters.

### 4.3. Processing Map

#### 4.3.1. The Analysis of Strain Rate Sensitivity Factor m

**Figure 4:**
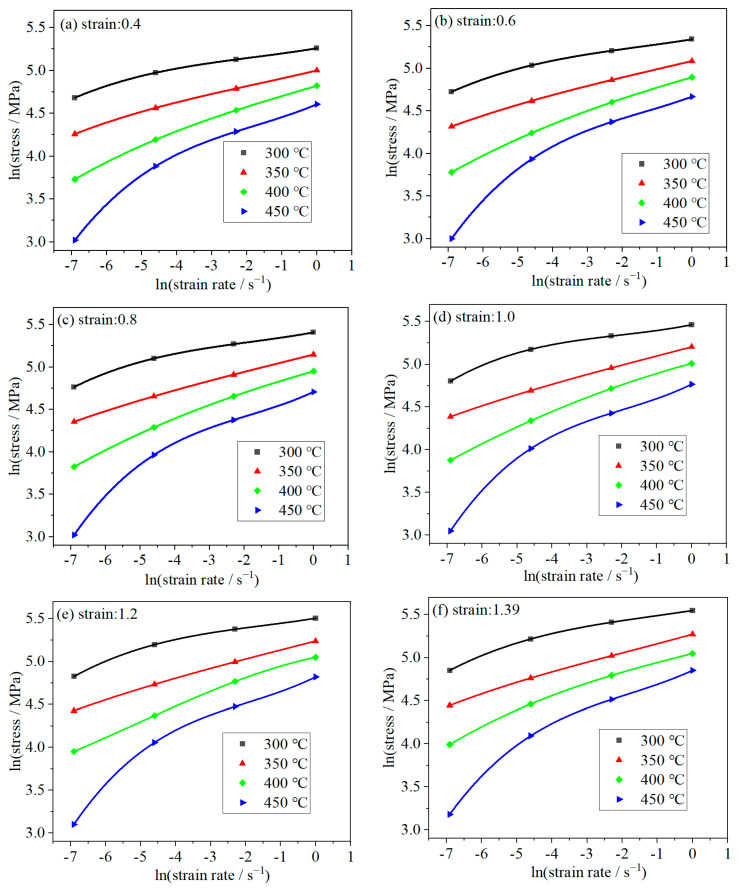
The relationship between ln(stress) and ln(strain rate) at different strains.

The processing map is very useful for guiding the hot working process. It shows both the power dissipation situation and the distribution of unstable areas. Thus, the power dissipation efficiency *η* and instability parameter $\xi (\dot{\varepsilon })$ should be obtained. However, in order to compute *η* and $\xi (\dot{\varepsilon })$, the strain rate sensitivity factor (*m*) under different conditions should be calculated in advance. The factor *m* plays a vital role in the interrelation between the flow stress and the processing map. According to Equation (2), *m* can be computed via the curving fitting of $\ln\sigma -\ln\dot{\varepsilon }$. Generally, a cubic spline function is employed for establishing the relationship between lnσ and $\ln\dot{\varepsilon }$ at a specified strain. It can be expressed as:

(8) $$\ln\sigma =a+b\ln\dot{\varepsilon }+c{\left(\ln\dot{\varepsilon }\right)}^{2}+d{\left(\ln\dot{\varepsilon }\right)}^{3}$$

where *a*, *b*, *c*, and *d* can be estimated via curving fitting. Figure 4 depicts the relationship between lnσ and $\ln\dot{\varepsilon }$ at various strains.

According to Equations (8) and (2), the following expression can be utilized to compute the factor *m*:

(9) $$m=b+2c\ln\dot{\varepsilon }+3d{\left(\ln\dot{\varepsilon }\right)}^{2}$$

**Figure 5:**
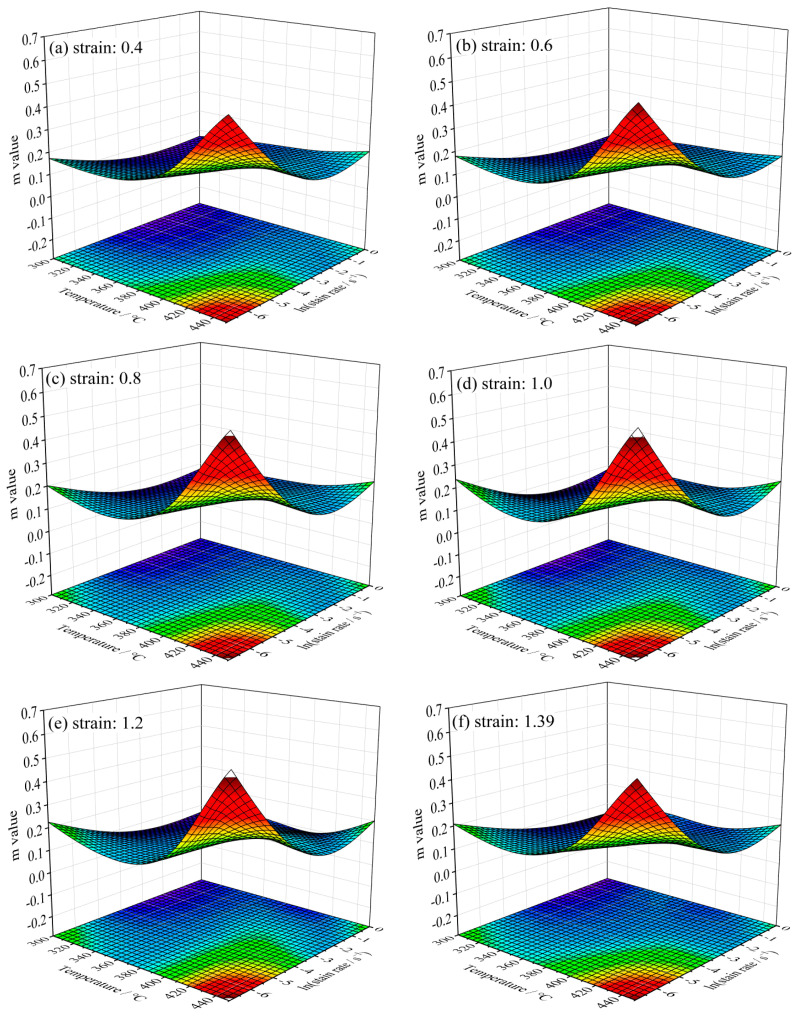
Influence of strain on strain rate sensitivity factor (*m*). The red color signifies a large *m* value, and the blue color manifests a low *m* value.

The calculated *m* values under four different temperatures and strain rates can be stored in a 4 × 4 matrix. In order to visualize the relationship between the deformation parameters and *m* value, the *m* value was interpolated, and then a 100 × 100 matrix was derived. Figure 5 displays the smooth 3D surfaces of the *m* value, in which the x-axis represents the temperature and y-axis represents the strain rate. Clearly, the *m* values alter irregularly with the deformation temperature and strain rate. However, the shapes of the surfaces at different strains are similar. The statistical results of the maximum and minimum *m* values are shown in Table 2. It can be seen that *m* value alters irregularly with the increase in strain.

#### 4.3.2. The Analysis of Power Dissipation Efficiency η

**Figure 6:**
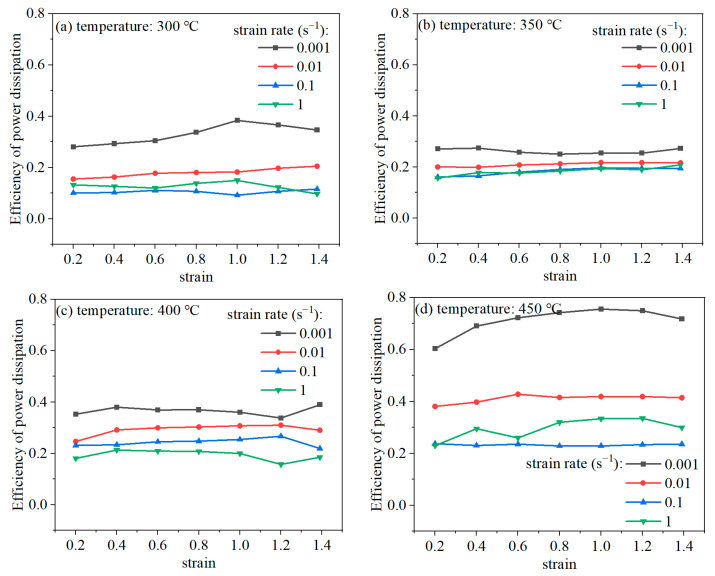
The relationship between power dissipation efficiency (*η*) and strain.

According to Equation (6), *η* can be calculated once the *m* values have been obtained. Figure 6 depicts the relationship between *η* and the strain. It can be clearly observed that the effects of the strain on *η* are different under various strain rates. When the strain rates are 0.001 and 1 s^−1^, *η* is highly dependent on the strain. During deformation at the strain rates 0.1 and 0.01 s^−1^, *η* is relatively unaffected by the strain. Also, it can be seen that the *η* value decreases with the increase in the strain rate at a constant deformation temperature.

**Figure 7:**
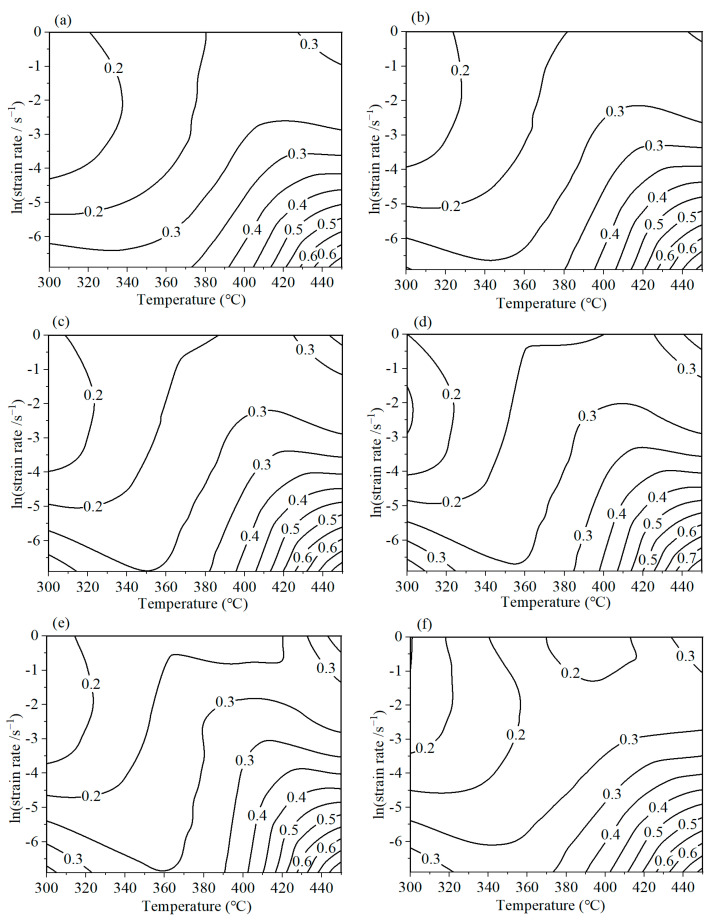
The power dissipation map of AA7075 under different strains: (**a**) 0.4; (**b**) 0.6 (**c**) 0.8; (**d**) 1.0; (**e**) 1.2; (**f**) 1.39.

Based on the above dissipation efficiency, a 2D dissipation map can be established. Figure 7 predicts the power dissipation situations of AA7075 when processing at different strains. The contour number indicates the dissipation efficiency. A certain relationship can be constructed between the dissipation efficiency and microstructure evolution mechanism. It can be observed that the maximum dissipation efficiencies surpass 0.6. Commonly, a higher dissipation efficiency is required. However, when processing in the higher-dissipation-efficiency regions, the product may also generate microstructure defects. Thus, in order to obtain the satisfactory mechanical properties of the alloys, the processing parameters should not be located in the flow instability areas.

#### 4.3.3. The Analysis of Instability Parameter ξ

Utilizing Equations (7) and (9), a calculated formula for the instability parameter *ξ* can be obtained:

(10) $$\xi (\dot{\varepsilon })=\frac{6d\ln\dot{\varepsilon }+2c}{m(m+1)\ln(10)}+m\leq 0$$

**Figure 8:**
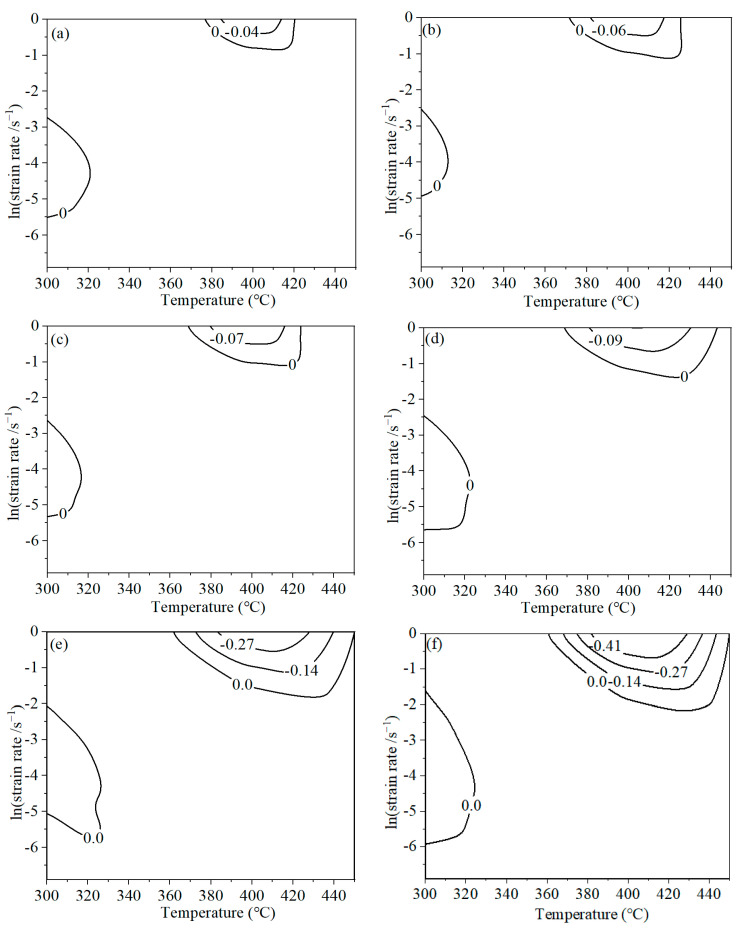
Instability map of AA7075 under different deformation condition with various strains. (**a**) 0.4; (**b**) 0.6 (**c**) 0.8; (**d**) 1.0; (**e**) 1.2; (**f**) 1.39.

Based on Equation (10), the *ξ* value can be estimated. Subsequently, the instability maps were constructed, as illustrated in Figure 8. When the compressed strain reaches 0.4, two instability domains exist. The instability domains are located at (375–420 °C, 0.419–1 s^−1^) and (300–320 °C, 0.004–0.064 s^−1^). For different strains, the shapes of the instability domains are similar. However, the areas of the instability region are different. When the strain increases from 0.4 to 1.39, the areas of the instability regions increase to 6.86%, 7.03%, 7.67%, 12.26%, 17.58%, and 20.54%, respectively.

#### 4.3.4. The Analysis of Processing Map

**Figure 9:**
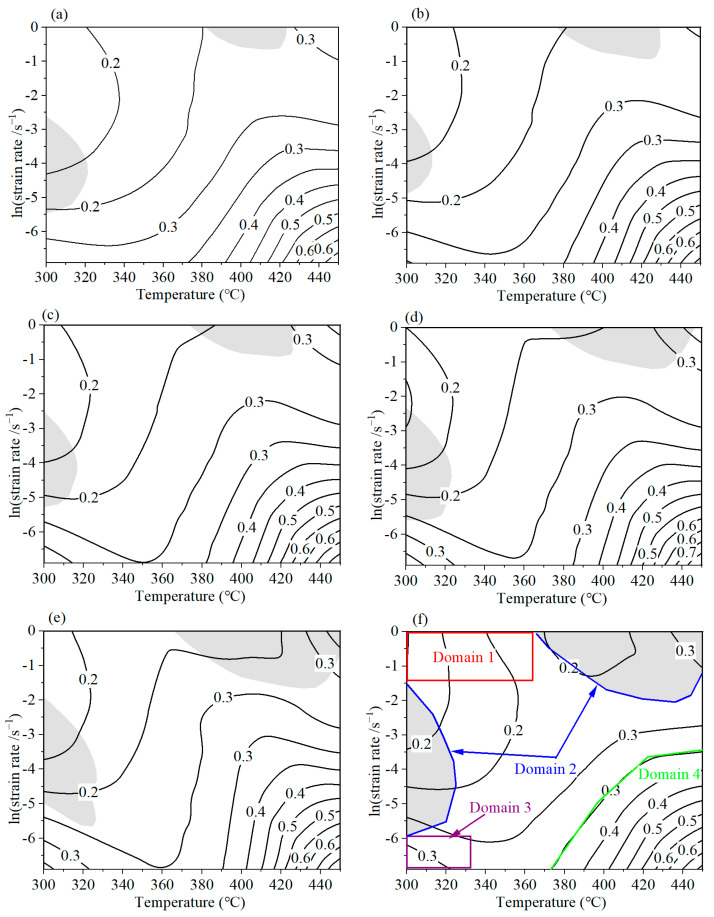
The processing maps of AA7075 under different strains: (**a**) 0.4; (**b**) 0.6 (**c**) 0.8; (**d**)1.0; (**e**) 1.2; (**f**) 1.39. (The values of the contour lines represent the power dissipation efficiency; the shaded area signifies the unstable processing region; domains 1, 2, 3, and 4 are four distinct processing regions according the power dissipation efficiency and instability parameter.).

A power dissipation map was obtained, which can contribute to identifying the high-dissipation-level zones. On the other hand, an instability map was also acquired, which is capable of avoiding the flow instability regions. Taking the comprehensive influence of these two kinds of maps, a processing map can be established [31]. Figure 9 displays the processing maps of AA7075 at various strains from 0.4 to 1.39. The contour number denotes the power dissipation efficiency; the grey shaded area indicates the negative instability parameter domain, which is the instability domain.

According to Figure 9, it can be observed that the maximum dissipation efficiencies at different strains are located in the domain with a higher temperature and lower strain rate, namely the right bottom corner of the processing map. And the maximum dissipation efficiencies are over 0.6 under various strains. At a strain of 1.39 (the corresponding engineering strain is 0.75), as shown in Figure 9f, four distinct domains have been partitioned. There are three stable domains and one unstable domain. Domain 2 is the unstable region, which occurs at (365–450 °C, 0.132–1 s^−1^) and (300–325 °C, 0.003–0.2332 s^−1^). In domain 1, the dissipation efficiency changes from 0.15 to 0.2, and it is a stable region. In domain 3, the dissipation efficiency increases to 0.3. This is identified as a stable region. Domain 4 occurs at (375–450 °C, 0.001–0.03 s^−1^), which owns the highest *η* value, ranging from 0.3 to 0.6. It is considered the optimum processing domain.

### 4.4. Microstructure Examination

**Figure 10:**
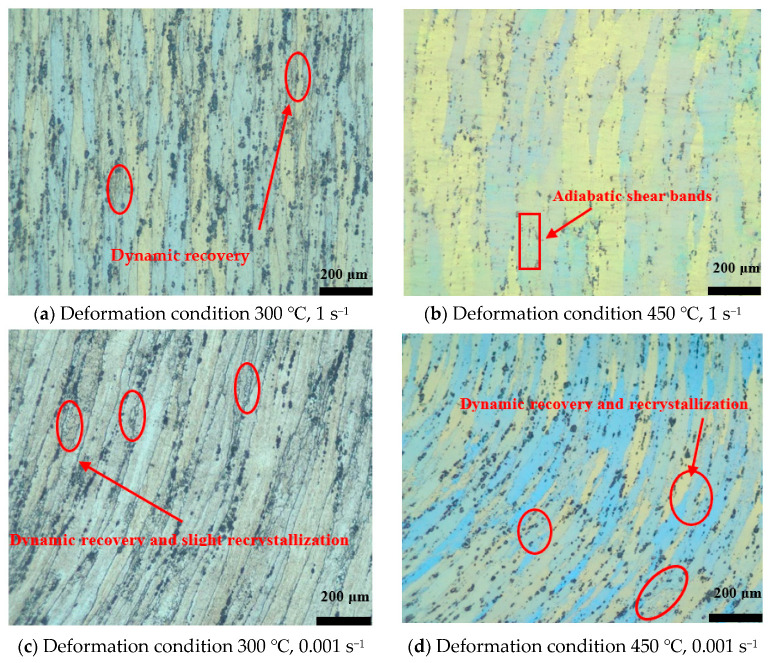
The OM observation of AA7075.

Figure 10 depicts the micrographs of AA7075 under different deformation parameters. After compression, the deformed grains still show a fibrous shape for all the test conditions. When the compression condition is 300 °C/1 s^−1^ (domain 1), the slenderness ratio of the fibrous grains decreases compared to that of the initial grain, as depicted in Figure 2a. This phenomenon originates from the fact that the compression and extrusion directions are parallel. In addition, there are more dynamic recovery structures, as manifested in domain 1, where the dominant softening mechanism is dynamic recovery. When the temperature increases to 450 °C (falling into domain 2), because of the generation of low-angle boundaries, the grain boundaries become blurry. In this situation, the formation of adiabatic shear bands can also be observed, as shown in Figure 10b. This microstructural characteristic is detrimental to obtaining a homogeneous grain structure. Thus, the hot working parameters should not be located within this domain. This conclusion is coincident with the results of the processing maps. When the strain rate decreases to 0.001 s^−1^ (domain 3), more dynamic recovery and a slight dynamic recrystallization structure can be found, as shown in Figure 10c. When the compression condition is 450 °C/0.001 s^−1^ (domain 4), the fibrous grains become shorter and finer compared to those in the other deformation conditions. Additionally, many dynamic recovery and recrystallization structures can be observed. Thus, it can be regarded as the optimum processing region in this study. However, there exists a possibility that if the compression temperature is higher or/and the strain rate is lower, a better grain structure can be obtained [32].

**Figure 11:**
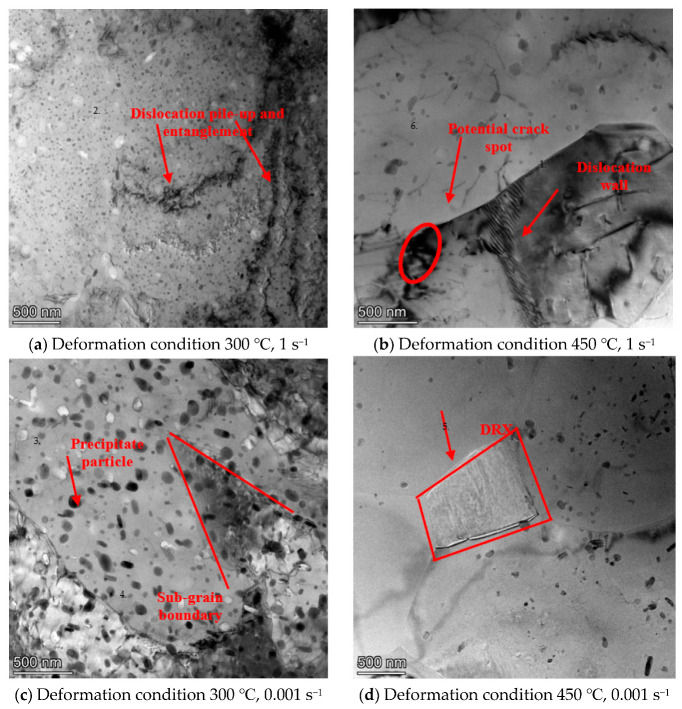
The TEM observation of AA7075.

Figure 11 illustrates the microstructure evolution of AA7075 under various deformation conditions observed via TEM. It can be observed that at 300 °C/1 s^−1^ (domain 1), grains of the compressed sample contain a high density of dislocation pile-up and entanglement, as depicted in Figure 11a. Under a low compression temperature, the deformation-induced driving force for the dislocation movement is insufficient, resulting in dislocation pile-up and the formation of dislocation entanglements [33,34,35].

When the deformation temperature increases to 450 °C (domain 2), the dislocations pile-up and entanglement decrease dramatically. This can be seen by comparing Figure 11a,b. The dislocation movability is enhanced because of the increase in temperature. Thus, the dislocations transform into dislocation lines and dislocation walls. In addition, there exists a highly dislocated region, which is formed due to the presence of particles in Figure 11b, as marked by a red circle. This region may be a potential crack and defect formation point. Because there is no ample time for dislocation migration and annihilation when it is deformed at a high strain rate, it will accumulate dislocations and result in stress concentration. Thus, at a high strain rate, the dislocations are prone to accumulate around the particle, which may act as a stress concentration point and nucleate the crack in domain 2. It is clear that domain 2 is the unstable area.

When the strain rate decreases from 0.001 s^−1^ (domain 3), the density of dislocation shows a decreasing trend, as depicted in Figure 11c. The reason is that, at a lower strain rate, dislocation movement occurs in sufficient time. Some sub-grain boundaries are observed, manifesting the presence of dynamic recovery.

When the sample is compressed at 450 °C/0.001 s^−1^ (domain 4), as shown in Figure 11d, the dislocations decrease dramatically, and a dynamic recrystallization (DRX) grain can be seen. During the formation process of DRX grains, the dislocation will rearrange and annihilate. This is conducive to reducing the stress concentration and forming a stable microstructure. Thus, a finer grain structure can be obtained when it is deformed in this region. 

Though only one hot working parameter was selected in each domain, the microstructure analysis matches the processing map results very well. This validates the effectiveness of the processing map. Thus, it can be concluded that, under a strain of 1.39, the optimum hot working domain is identified at (375–450 °C, 0.001–0.03 s^−1^), in which the corresponding peak efficiency is 0.6.

## 5. Conclusions

Compression tests under four temperatures (300, 350, 400, and 450 °C) and four strain rates (0.001, 0.01, 0.1, and 1 s^−1^) were performed using a Gleeble-3500 isothermal simulator. The hot workability of 7075 aluminum alloy was investigated using a processing map and microstructural observation. The main conclusions are as follows:

1. The flow stress level of 7075 aluminum alloy under isothermal compression increased significantly with an increasing strain rate or decreasing deformation temperature.
2. In the construction of the processing map, the determination of the instability region should consider both the negative dissipation efficiency and instability parameters.
3. The microstructure analysis matches the processing map results very well. Based on the processing maps, the optimum hot processing domain of 7075 aluminum alloy at a strain of 1.39 is located at (375–450 °C, 0.001–0.03 s^−1^), in which the maximum efficiency reaches 0.6. The most unfavorable areas are located at (365–450 °C, 0.132–1 s^−1^) and (300–325 °C, 0.003–0.2332 s^−1^), which should be avoided in the hot working process.

## Figures and Tables

**Table 1:** The chemical composition of the studied alloy.

Composition	Zn	Mg	Cu	Si	Cr	Fe	Mn	Ti	Al
Content (wt.%)	5.8	2.3	1.5	0.07	0.21	0.16	0.05	0.02	Bal.

**Table 2:** Effect of strain on the maximum and minimum *m* values.

**True Strain**	0.4	0.6	0.8	1.0	1.2	1.39
**Maximum *m* value**	0.5269	0.5652	0.5897	0.6073	0.5991	0.5598
**Minimum *m* value**	0.0540	0.0590	0.0567	0.0484	0.0567	0.0512

## Data Availability

Data are contained within the article.
